# MetabNet: An R Package for Metabolic Association Analysis of High-Resolution Metabolomics Data

**DOI:** 10.3389/fbioe.2015.00087

**Published:** 2015-06-11

**Authors:** Karan Uppal, Quinlyn A. Soltow, Daniel E. L. Promislow, Lynn M. Wachtman, Arshed Ali Quyyumi, Dean P. Jones

**Affiliations:** ^1^Division of Pulmonary Medicine, Department of Medicine, Emory University, Atlanta, GA, USA; ^2^Department of Pathology, University of Washington, Seattle, WA, USA; ^3^New England Primate Research Center, Harvard University, Southborough, MA, USA; ^4^Division of Cardiology, Department of Medicine, Emory University, Atlanta, GA, USA

**Keywords:** metabolomics, metabolite identification, metabolic networks, metabolic pathways, targeted MWAS, choline

## Abstract

Liquid-chromatography high-resolution mass spectrometry provides capability to measure >40,000 ions derived from metabolites in biologic samples. This presents challenges to confirm identities of known chemicals and delineate potential metabolic pathway associations of unidentified chemicals. We provide an R package for metabolic network analysis, MetabNet, to perform targeted metabolome-wide association study of specific metabolites to facilitate detection of their related metabolic pathways and network structures.

## Introduction

Metabolomics using high-resolution mass spectrometry coupled to liquid chromatography (LC/MS) provides a practical approach to detail physiological chemistry for personalized medicine (Johnson et al., 2010; Soltow et al., 2013; Uppal et al., 2013). Advances have occurred at all levels in instrumentation, standard operating procedures, and data extraction and analysis (Chen et al., 2012; Ivanisevic et al., 2013; Uppal et al., 2013). Current capabilities at Emory University allow detection of >40,000 ions within individual samples and >100,000 ions within population studies. The number of ions detected depends upon the stringency of the data extraction parameters; many of the ions have relatively poor coefficient of variability and/or are present in only a small number of samples. Presently, data are filtered to much smaller numbers for statistical analyses. In principle, however, the spectrum of chromatographic methods and detection techniques could allow routine quantification of even more chemicals if systematically applied and appropriately curated. Such development would have considerable utility for study of complex mechanisms of human disease involving diet, environmental exposures, microbiome, and health behaviors (Jones et al., 2012). Application in a systematic way would also provide an approach to elucidate impact of cumulative lifetime exposures, termed the exposome (Wild, 2005, 2012; Miller and Jones, 2013).

**Figure 1:**
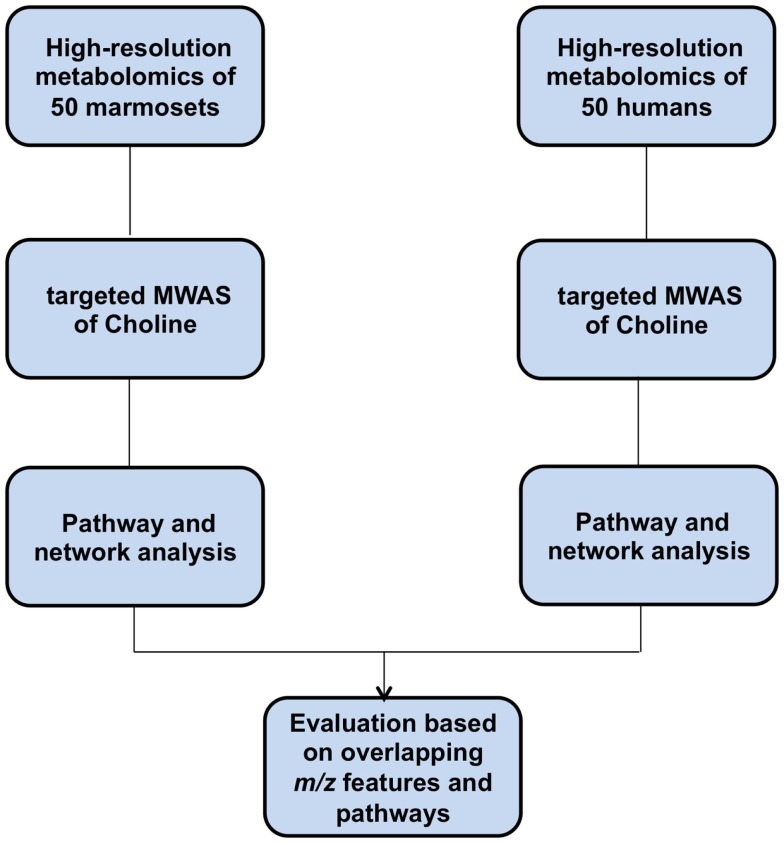
**Overview of the study design**.

Metabolome-wide association studies (MWAS), illustrated as Manhattan plots of the negative log of the *p*-values for association of each metabolite with a parameter of interest, are useful for characterization of metabolites associated with disease or experimental manipulation (Osborn et al., 2013; Go et al., 2014). We use the term “targeted MWAS” to refer to metabolome-wide associations with a specific known chemical target. The purpose of this paper is to describe an R package, MetabNet, to facilitate use of targeted MWAS for pathway and network mapping. To illustrate the logic and use of MetabNet, we selected choline, an important precursor for phosphatidylcholines and a dietary precursor for 1-carbon metabolism linked to cardiovascular disease (Tang et al., 2013), as a useful example. A schematic overview of the study design is presented in Figure 1.

## Materials and Methods

### Plasma from common marmosets (*Callithrix jacchus*)

Data for the present study were derived from plasma samples from 50 healthy marmosets, aged 2–14 years, selected from ongoing aging studies of marmosets (Roede et al., 2013); the samples included an equal number of males and females. Animal care was as described (Soltow et al., 2013). Briefly, marmosets were housed at the New England Primate Research Center and fed commercial food (New World Primate Chow 8791, Harlan Teklad, Indianapolis, IN, USA) supplemented with a combination of fresh fruits, vegetables, seeds, eggs, and/or mealworms. Fresh water was provided *ad libitum*. EDTA plasma was prepared from blood collected under ketamine anesthesia during routine physical examination and stored at −80°C before LC/MS analysis. The facility is AAALAC-accredited, and all works were approved by Harvard Medical School’s Standing Committee on Animals.

### Plasma from healthy humans

Data for the present study were derived from EDTA plasma samples from 50 healthy humans analyzed as part of an ongoing healthy aging study (A.A. Quyyumi, Emory IRB protocol # IRB00024767). The study included individuals between 30 and 90 years, with even distribution of sex, and included individuals of different races and ethnicities present in the Atlanta area. All individuals were extensively screened to assure good health in terms of the absence of disease markers and absence of therapeutic drug use. Because the samples were de-identified and analysis was randomized and blinded, no additional details are available. Plasma was stored at −80°C before LC/MS analysis.

### High-resolution metabolomics

Samples were extracted and analyzed as previously described (Soltow et al., 2013; Uppal et al., 2013). Briefly, extractions were performed with acetonitrile containing a mixture of internal standards and maintained in an autosampler maintained at 4°C until injection. Liquid chromatography was performed using a C18 column (Higgins Analytical, Targa, 2.1 × 10 cm) with a short, end-capped C18 pre-column (Higgins Analytical, Targa guard) and an acetonitrile gradient. Mass spectrometry was performed using an LTQ-Velos-Orbitrap mass spectrometer (Thermo Fisher, San Diego, CA, USA): HESI probe with S-lens combination for ESI; MS1 mode scanning *m/z* range of 85–2000; resolution, 60,000; maximum number of ions collected, 5.00 × 10^5^; the maximum injection time, 5 μL/s; capillary temperature, 275°C; source heater, 45°C; voltage, 4.6 kV; sheath gas, 45; auxillary gas flow, 5; sweep gas flow, 0. Each sample was run in triplicate with 10 μL injection volume. Data were collected continuously over the 10-min chromatographic separation and stored as .raw files. These files were converted using XCalibur file converter software (Thermo Fisher, San Diego, CA, USA) to .cdf files for further data processing. The .raw files are time and date stamped and electronically archived as the original data for use in subsequent reanalysis as necessary. The data were processed for peak extraction and quantification of ion intensities using xMSanalyzer software (Uppal et al., 2013) with apLCMS (Yu et al., 2009). Feature and sample filtering retained features that had a median CV <50%, a Pearson correlation >0.7 among technical replicates, and <30% missing values. Features were annotated by searching Metlin with *m/z* tolerance of 10 ppm. Correlation analysis was performed using Pearson’s correlation method. False discovery rate correction was performed using the Benjamini and Hochberg procedure (Benjamini and Hochberg, 1995). Both .raw and .cdf files have been submitted to the NIH Metabolomics Workbench repository.

## Results

**Figure 2:**
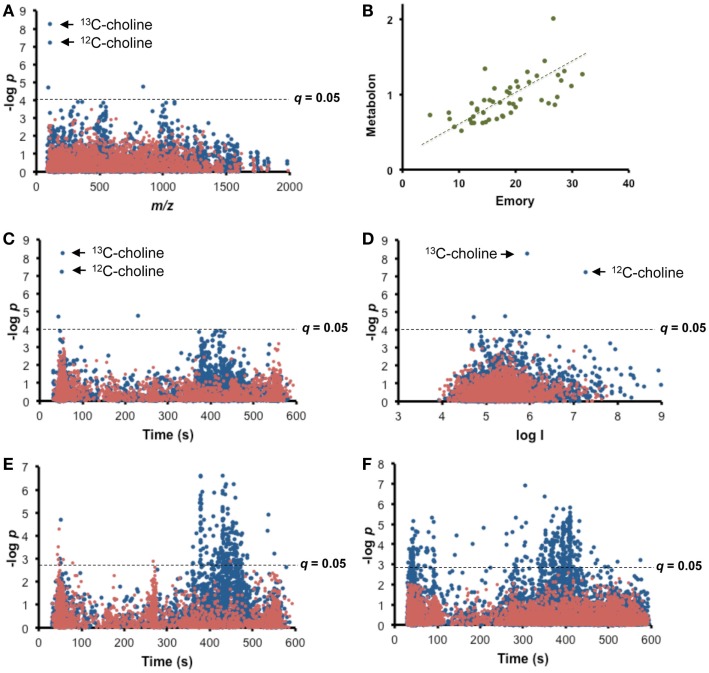
**Targeted MWAS for choline**. **(A)** Type 1 Manhattan plot for Pearson correlation of targeted choline measurements (Metabolon) with each *m/z* feature in high-resolution metabolomics (HRM) for 50 plasma samples from common marmosets. **(B)** Comparison of Metabolon values with corresponding HRM analysis at Clinical Biomarkers Laboratory. Values are expressed as relative units. **(C)** Type 2 Manhattan plot of targeted choline measurements as a function of retention time of *m/z* feature suggests association of choline with lipids. **(D)** Type 3 Manhattan plot of targeted choline measurements as a function of ion intensity shows that correlated ^12^C and ^13^C forms of choline have expected differences in ion intensity and that other significantly associated and unidentified *m/z* features are present with lower ion intensities. **(E)** Internal Targeted MWAS is shown as type 2 Manhattan plot for Pearson correlation of choline within the HRM analysis. Results show significant associations of choline with *m/z* features eluting after 350 s. **(F)** Targeted MWAS for choline in human plasma. Type 2 Manhattan plot for Pearson correlation of choline within the HRM analysis of plasma of 50 healthy humans shows significant associations of choline *m/z* features eluting after 350 s. Mean choline concentration was 5.8 ± 0.8 μM. In (**E)** and (**F)**, self-correlations of different forms of choline were removed to facilitate visualizations of correlation of choline with other *m/z* features. Positive correlations are shown in blue and negative correlations in red. FDR corrected significance level is shown as broken line.

Fifty plasma samples from common marmosets (*Callithrix jacchus*) were sent to a commercial laboratory (Metabolon, Durham, NC, USA) for measurement of choline. A targeted MWAS for plasma choline (Figure 2A) was obtained by testing for correlation of these targeted choline measurements with the ion intensity for each *m/z* feature among the 5407 measured by high-resolution metabolomics (HRM; Soltow et al., 2013; Uppal et al., 2013) in the same samples at the Emory facility (Figure 2A). Results showed that two *m/z* features (*m/z* 105.1095, 53 s; *m/z* 104.1062, 51 s) in HRM, matching the ^13^C and ^12^C forms of choline [M + H]^+^, respectively, had high Pearson correlations (*r* = 0.72, 0.68, respectively) with the Metabolon data, and were significant at FDR <0.05 (raw *p*-values < 10^−7^). MS/MS confirmed identification of *m/z* 104.1062 as choline (data not shown). Two other features that significantly correlated with Metabolon data were not readily identifiable (841.9269 had no matches in Metlin, and 361.1802 matched 12 tripeptides as [M + H]^+^, 18 tripeptides as [M + 2Na-H]^+^, an insecticide and natural products as [M + Na]^+^, and a plant-derived diterpenoid as [M-H_2_O]^+^). A direct plot of the Metabolon values with the intensity for *m/z* 104.1062 (Figure 2B) illustrates concurrence of the methods. Thus, this simple approach provides a convenient means for cross-validation of platforms and conversion of an untargeted HRM platform into a hybrid platform in which *m/z* features matched to metabolites in database searches are verified by significant correlation with authenticated chemical analysis.

Re-plotting the significance test data as a function of retention time, which we term a “type 2” Manhattan plot (Figure 2C), yields information on chromatographic properties of the correlated *m/z* features. A similar “type 3” Manhattan plot as a function of ion intensity visualizes the relationship of the abundance of signals for ^13^C and ^12^C forms of choline (Figure 2D). Importantly, in the type 2 Manhattan plot, many *m/z* features that elute after 350 s are weakly correlated with choline even though choline eluted at 52 s. This is more directly visualized by performing an internal targeted MWAS in which *m/z* 104.1062 [choline + H]^+^ is regressed against other *m/z* features within the same analysis (Figure 2E), thereby eliminating inter-laboratory, cross-platform differences. With this analysis, 198 features significantly associated (FDR <0.05) with choline (Supplementary Results, Table S1 in Supplementary Material). Many lipids elute after 350 s under these chromatographic conditions suggestive of associations of choline with blood lipids. Correlated *m/z* feature matches included phosphocholine (*m/z* 184.0719 [M + H]^+^), glycerophosphocholine (*m/z* 258.1083 [M + H]^+^), and a number of phosphatidylcholines, some detected in multiple forms (Table 1). Out of 42 with matches to [M + H]^+^ in Metlin, 18 matched phospholipids, including 6 ceramides and other complex lipids, and 3 matched tripeptides. Importantly, 156 of the 198 *m/z* features did not have Metlin database matches despite the extensive nature of this database, reinforcing the contemporary challenges to curation of the metabolome.

**Table 1:** **Selected *m/z* features correlated (FDR <0.05) with choline in targeted MWAS**.

Match	Adduct	*m/z*	Retention time (s)	*r*	Log intensity
**Fifty marmoset samples**
Phosphocholine	[M + H]^+^	184.0719	417	0.55	7.16
Glycerophosphocholine	[M + H]^+^	258.1083	426	0.46	5.84
PC(16:1/0:0)	[M + H]^+^	494.3207	378	0.65	7.94
PC(16:1/0:0)	[M-H_2_O]^+^	476.3099	380	0.61	6.22
PC(16:1/0:0)	[M + Na]^+^	516.3030	379	0.58	6.45
^13^C-PC(16:1/0:0)	[M + Na]^+^	517.3063	377	0.61	5.83
PC(16:0/0:0)	[M + H]^+^	496.3376	442	0.59	8.93
PC(18:1/0:0)	[M + H]^+^	522.3532	460	0.54	8.60
**Fifty human samples**
Phosphocholine	[M + H]^+^	184.0724	381	0.57	5.23
Glycerophosphocholine	[M + H]^+^	258.1089	378	0.55	5.75
PC(16:1/0:0)	[M + H]^+^	494.3239	354	0.54	5.28
PC(16:1/0:0)	[M-H_2_O]^+^	476.3103	360	0.53	6.09
PC(16:1/0:0)	[M + Na]^+^	516.3047	351	0.64	5.13
^13^C-PC(16:1/0:0)	[M + Na]^+^	517.3065	351	0.47	5.80
PC(16:0/0:0)	[M + H]^+^	496.3358	395	0.55	5.44
PC(18:1/0:0)	[M + H]^+^	522.3513	410	0.58	5.49

To test generalization of the targeted MWAS approach for detection of metabolic associations, HRM data from 50 healthy human subjects were examined for the apparent pathway associations detected in marmosets. The results showed that the ^13^C form of choline (*m/z* 105.1097 [M + H]^+^, 42 s) correlated with *m/z* 104.1064 (44 s; *r* = 0.96; *p* = 1.5 × 10^−19^) in the human samples. Significant *m/z* features (Table S2 in Supplementary Material) were present, which correlated with choline and matched the same adducts in Metlin, as obtained for the marmoset samples (Table 1).

**Figure 3:**
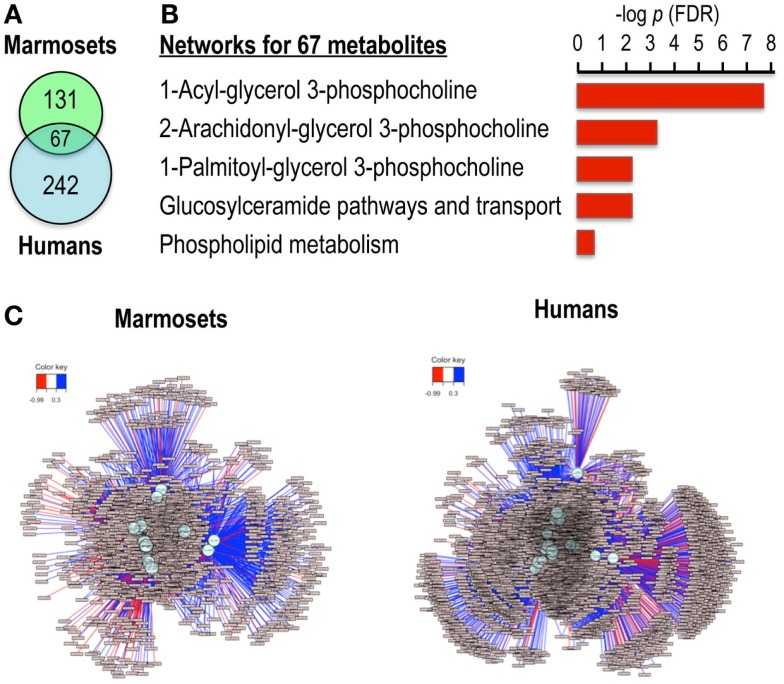
**Ions correlated with choline in both marmoset and human samples map to phosphocholine pathways in network enrichment analysis**. **(A)** Venn diagram shows 67 ions were common among the 198 correlated ions for marmosets and 309 correlated ions for humans. **(B)** Analysis of these 67 ions by MetaCore showed significant pathway enrichments after FDR correction for multiple phosphocholine pathways. **(C)** Metabolome-wide network structure. Correlation analyses showed that 18 of the 67 ions had similar pairwise correlation patterns for marmosets and humans at *p* < 0.05. These were tested for correlations among other ions, and features that had absolute Spearman correlation >0.3 at FDR <0.05 are shown for marmosets and humans, demonstrating ability to map the broader network structure associated with choline. Stratification according to strengths of associations showed that the most highly associated clusters contained phospholipids, while terpenoids and steroids were present in less strongly associated clusters.

Comparison of the significant features for the marmoset and human data using the getVenn function in xMSanalyzer (Uppal et al., 2013) showed that 67 *m/z* features were common to both species (Figure 3A). Pathway enrichment analyses for these 67 features using MetaCore (Thomson Reuters; https://portal.genego.com/) showed significant enrichment of phosphocholine-related lipid pathways (Figure 3B). A correlation test at significance level of 0.05 was performed to evaluate the similarity of the pairwise correlation patterns of the 67 features between the two species. This resulted in detection of 18 features common to both species that were significantly correlated with choline and also had similar expression patterns in the two species. Subsequently, association networks derived from the Spearman correlations of these metabolites with others (|*r*| > 0.3; *q* < 0.05) were generated to map the direct and indirect associations of choline in both species (Figure 3C). Phospholipids were found in clusters more highly associated with choline than clusters containing terpenoids and steroids. Thus, the results show that targeted MWAS can be useful to map metabolic pathway associations, facilitating curation of the metabolome.

## Discussion

Targeted MWAS does not replace MS^n^ or other targeted analysis for verification of identity of chemicals. The method does, however, provide a useful complement to (1) detect multiple forms of a chemical (e.g., ^12^C and ^13^C forms of choline), (2) verify quantification by different platforms, (3) associate unidentified ions with established metabolic pathways and (4) map metabolic network structure with a target of interest. This complements other approaches to map metabolic networks (Kanehisa et al., 2002; Li et al., 2013) for curation of the metabolome.

The procedures of targeted MWAS and generation of panels for Figure 2 involved commonly available software (Excel, Microsoft). Similarly, data annotation and pathway mapping used readily available and easy-to-use online resources. Consequently, the approach can be rapidly assimilated into metabolomics research, allowing translation of older targeted metabolomics methods to studies using contemporary high-resolution mass spectrometers. Alternatively, we have incorporated the functionality to perform targeted MWAS and generate correlation-based networks in an R package, MetabNet, which is available at: https://sourceforge.net/projects/metabnet/. The package can be used to generate full or partial correlation networks. Partial correlation network analysis, where the correlation between two variables is adjusted by taking into account the effect of other variables, is performed using the *cor2pcor* function in the corpcor R package. (Schäfer and Strimmer, 2005; Opgen-Rhein and Strimmer, 2007) The metabnet function in the MetabNet package can be used to perform full or partial correlation network analysis. The main input arguments are described below:

*feature_table_file*: Feature table that includes the *m/z*, retention time, and measured intensity in each sample. The first two columns should be the *m/z* and time. The remaining columns should correspond to the samples in the class labels file with each column including the intensity profile of a sample. Full path required, e.g., C:/My Documents/test.txt. The feature table should be in a tab-delimited format. An example input file is provided on the project website: https://sourceforge.net/projects/metabnet/files/Tutorial_files/.
*target.metab.file*: File that includes the *m/z* and/or retention time of the targeted *m/z* features corresponding to metabolites of interest. The first column should contain the *m/z* of the features. Alternatively, retention time information could be provided in the second column to search by both *m/z* and time. The file should be in a tab-delimited format.
*sig.metab.file*: (Optional) file with list of *m/z* of the discriminatory features, e.g., features that are significantly different between case vs control, to perform targeted MWAS with these features. Default value: NA.
*class_labels_file*: (Optional) File with class/group information for each sample. Samples should be in the same order as in the feature table. Default value: NA.

### Output

The program generates a PDF file with network plots and also writes a network file in the GML format that can be used as input for Cytoscape (Shannon et al., 2003) to facilitate interactive visualization and downstream network analysis. The package generates text files. Currently, the package does not automatically generate output compatible with data repositories; however, an Rda file is generated that includes all the user-defined options, input file names and data matrices, and output correlation matrix as R objects. This allows users to retrieve the input options used for network analysis for analysis reproducibility. Furthermore, users can use the R objects in the Rda file to generate the output matrix according to requirements of public data repositories without re-running the analysis.

More information can be found in the package manual available at: http://sourceforge.net/projects/metabnet/files/MetabNet-manual.pdf. A tutorial with installation and usage instructions is provided as Supplementary Material (Data Sheet S1 in Supplementary Material).

**Figure 4:**
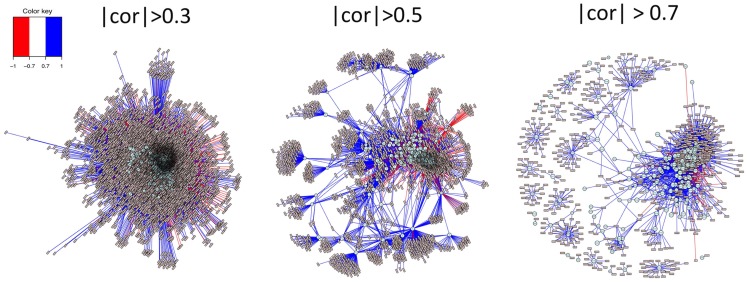
**Systematic variation in stringency of correlation allows visualization of primary and secondary network structures**. Analysis of ions positively and negatively correlated with a metabolite, as shown in Figure 2, aid in identification of unknown ions, especially when there are many database matches to the ion. By including secondary correlations in the analysis, as shown in Figure 3, one can begin to see a broader network organizational structure. This functionality is facilitated in MetabNet by providing ability to readily examine this network structure at different stringencies. At low stringency (|*r*| > 0.3), a large number of secondary correlations with choline are apparent. At greater stringency (|*r*| > 0.5), a number of secondary networks are evident in which many ions are significantly correlated with ions that are correlated with choline. This provides a basis to study perturbation in association with diet, genetic variation, and other causes of disease. Even higher stringency (|*r*| > 0.7) is useful in some cases to simplify the network structure to a small number of strong associations and also to facilitate identification of multiple ions, adducts, or isotopes derived from chemicals in the target list.

Functionality of MetabNet to map out metabolic network structure is illustrated in Figure 4. In this example, the stringency of correlation is systematically varied to visualize the networks of ions associated with choline, and the secondary network of metabolites correlated with those significantly correlated with choline. At low stringency (|*r*| > 0.3), large numbers of ions are correlated, most with positive correlation (blue lines) and a smaller number with negative correlation (red lines). At higher stringency (|*r*| > 0.5), secondary clusters of metabolites are clearly evident, providing a basis to study secondary metabolic processes that are linked to metabolites correlated with choline. This visualization can be important, for instance, in studies to evaluate effects of dietary or genetic variations of choline metabolism. Higher stringency (|*r*| > 0.7) further limits the number of correlations and simplifies initial characterization. High stringencies are also useful for ion identification because high positively correlated features that are also co-eluting often represent different ions derived from the same chemical as shown in recent studies. (Alonso et al., 2011; Lynn et al., 2015) This capability is useful to discriminate metabolites with extensive interactions with other metabolites, e.g., central energy metabolites, from those with little interaction, e.g., environmental or dietary components.

## Conclusion

MetabNet provides a convenient way to use targeted MWAS to improve curation of the metabolome and map out pathway and network structures. Human plasma contains a complex mixture of chemicals derived from diet, microbiome, infectious agents, pharmaceuticals, commercial products, and the environment. At current levels of detection using high-resolution mass spectrometry, 40,000 ions are detected; in principle, this number may be increased with improved separation, detection, and computational methods. MetabNet can be useful to simplify data analysis and enhance the utility of this high-resolution data by improved documentation of chemical identities and discovery of network structures and substructures that contribute to disease risk and responses to therapies.

## Conflict of Interest Statement

The authors declare that the research was conducted in the absence of any commercial or financial relationships that could be construed as a potential conflict of interest.

## Supplementary Material

The Supplementary Material for this article can be found online at http://journal.frontiersin.org/article/10.3389/fbioe.2015.00087/abstract
