# Supplementary material for: MetabNet: An R Package for Metabolic Association Analysis of High-Resolution Metabolomics Data
Source: Front Bioeng Biotechnol. 2015 Jun 11;3:87. doi: 10.3389/fbioe.2015.00087 (PMC4464066; doi:10.3389/fbioe.2015.00087)
Supplement: Supplementary file 1 [file data_sheet_1.docx]

***Supplementary Material***

**MetabNet: an R package for metabolic network analysis of high-resolution metabolomics data**

**Karan Uppal^1^, Quinlyn A Soltow^1^, Daniel E L Promislow^2^, Lynn Wachtman^3^, Arshed A Quyyumi^4^, Dean P Jones^1*^**

^1^Department of Medicine, Division of Pulmonary Medicine, Emory University, Atlanta, GA, USA.

^2^Department of Pathology, University of Washington, Seattle WA USA.

^3^New England Primate Research Center, Harvard University, Southborough, MA USA.

^4^Department of Medicine, Division of Cardiology, Emory University, Atlanta, GA USA.

*** Correspondence:** Dean P. Jones, Clinical Biomarkers Laboratory, Division of Pulmonary, Allergy and Critical Care Medicine, Department of Medicine, Emory University, 205 Whitehead Research Center, Atlanta, GA 30322, USA dpjones@emory.edu

**1. MetabNet installation (Availability:** https://sourceforge.net/projects/metabnet/)

| a) R commands for installing dependencies: | | | | |  |  |  |  |  |  |  |  |
| --- | --- | --- | --- | --- | --- | --- | --- | --- | --- | --- | --- | --- |
|  |  |  |  |  |  |  |  |  |  |  |  |  |
| ##############START####################### | | | | |  |  |  |  |  |  |  |  |
| source("http://bioconductor.org/biocLite.R") | | | | |  |  |  |  |  |  |  |  |
| install.packages("WGCNA",repos="http://cran.r-project.org",dependencies=TRUE) | | | | | | | |  |  |  |  |  |
|  | | | | | | |  |  |  |  |  |  |
| install.packages("fdrtool",repos="http://cran.r-project.org") | | | | | |  |  |  |  |  |  |  |
| install.packages("doSNOW",repos="http://cran.r-project.org") | | | | | | |  |  |  |  |  |  |
| install.packages("mixOmics",repos="http://cran.r-project.org") | | | | | | |  |  |  |  |  |  |
| install.packages("statmod",repos="http://cran.r-project.org") | | | | | |  |  |  |  |  |  |  |
| install.packages("gplots",repos="http://cran.r-project.org") | | | | | |  |  |  |  |  |  |  |
| install.packages("rgl",repos="http://cran.r-project.org") | | | | | |  |  |  |  |  |  |  |
| biocLite("qvalue",suppressUpdates=TRUE)  biocLite("pcaMethods") | | | | |  |  |  |  |  |  |  |  |
| install.packages("corpcor",repos="http://cran.r-project.org") | | | | | |  |  |  |  |  |  |  |
| ##############END####################### | | | | |  |  |  |  |  |  |  |  |
|  |  |  |  |  |  |  |  |  |  |  |  |  |
|  |  |  |  |  |  |  |  |  |  |  |  |  |
| b) Install MetabNet |  |  |  |  |  |  |  |  |  |  |  |  |
|  |  |  |  |  |  |  |  |  |  |  |  |  |
| Installation on Windows: | | |  |  |  |  |  |  |  |  |  |  |
| 1) Download the "MetabNet_*.zip" file from https://sourceforge.net/projects/metabnet/ | | | | | | | | | | | |  |
| 2) Open R | |  |  |  |  |  |  |  |  |  |  |  |
| 3) Click on "Packages" under the menu bar | | | | |  |  |  |  |  |  |  |  |
| 4) Click on "Install packages from local zip files" | | | | |  |  |  |  |  |  |  |  |
| 5) Browse to the download location of the "MetabNet_*.zip" file | | | | | | |  |  |  |  |  |  |
| 6) Double click on the file and installation should begin | | | | | |  |  |  |  |  |  |  |
| 7) Run "library(MetabNet)" command from within R to make sure the package | | | | | | | |  |  |  |  |  |
| is successfully installed | | |  |  |  |  |  |  |  |  |  |  |
|  |  |  |  |  |  |  |  |  |  |  |  |  |
| MetabNet installation on Mac: | | |  |  |  |  |  |  |  |  |  |  |
| 1) Go to “Applications” | | |  |  |  |  |  |  |  |  |  |  |
| 2) Open R | |  |  |  |  |  |  |  |  |  |  |  |
| 3) Go to "Packages & Data” | | |  |  |  |  |  |  |  |  |  |  |
| 4) Select "Local Source Package" option from the drop down menu | | | | | | |  |  |  |  |  |  |
| 5) Click on "Install” | |  |  |  |  |  |  |  |  |  |  |  |
| 6) Browse to the download location | | | |  |  |  |  |  |  |  |  |  |
| 7) Click "Open" | |  |  |  |  |  |  |  |  |  |  |  |
|  |  |  |  |  |  |  |  |  |  |  |  |  |
| OR: |  |  |  |  |  |  |  |  |  |  |  |  |
|  |  |  |  |  |  |  |  |  |  |  |  |  |
| 1) Download "MetabNet_*.tar.gz" file from https://sourceforge.net/projects/metabnet/ | | | | | | | | | | | |  |
| 2) Go to MAC “Utilities” | | |  |  |  |  |  |  |  |  |  |  |
| 3) Click on "Terminal" | | |  |  |  |  |  |  |  |  |  |  |
| 4) Browse to the download location of MetabNet_*.tar.gz | | | | | |  |  |  |  |  |  |  |
| 5) Run "R CMD INSTALL MetabNet_*.tar.gz" to install | | | | | |  |  |  |  |  |  |  |
| 6) Run "library(MetabNet)" command from within R to make sure the package | | | | | | | |  |  |  |  |  |
| is successfully installed | | |  |  |  |  |  |  |  |  |  |  |
|  |  |  |  |  |  |  |  |  |  |  |  |  |
|  |  |  |  |  |  |  |  |  |  |  |  |  |
| MetabNet installation on Linux: | | | |  |  |  |  |  |  |  |  |  |
| 1) Download "MetabNet_*.tar.gz" file from https://sourceforge.net/projects/metabnet/ | | | | | | | | | | | |  |
| 2) Browse to the download location of MetabNet_*.tar.gz | | | | | |  |  |  |  |  |  |  |
| 3) Run "R CMD INSTALL MetabNet_*.tar.gz" to install | | | | | |  |  |  |  |  |  |  |
| 4) Run "library(MetabNet)" command from within R to make sure the package | | | | | | | |  |  |  |  |  |
| is successfully installed | | |  |  |  |  |  |  |  |  |  |  |

**2. Using MetabNet in R (Please refer to the manual for details on each argument):**

#load package

library(MetabNet)

###Change file locations#######

#1) complete peak intensity table (m/z and time). Please see example file for reference available at: http://sourceforge.net/projects/metabnet/files/

feature_table_file<-"C:/Users/ Documents/input_feature_table.txt"

#2) targeted m/z features

target_metab_file<-"C:/Users/ Documents/target_list.txt"

#3) optional: list of discriminatory features. Please see example file for reference.

sig_feat_file<-NA

#4) output location

outloc<-"C:/Users/ Documents/MetabNet_results/

################################

#allow use of multiple threads

allowWGCNAThreads()

net_res<-metabnet(feature_table_file=feature_table_file,

target.metab.file=target_metab_file, sig.metab.file=sig_feat_file,

parentoutput_dir=outloc,

class_labels=NA,cor.method="pearson",abs.cor.thresh=0.4,cor.fdrthresh=0.05,

cor.fdrmethod="BH",

target.mzmatch.diff=10,

target.rtmatch.diff=NA,max.cor.num=150,num_replicates=1,summarize.replicates=TRUE,

all.missing.thresh=0.8, rep.max.missing.thresh = 0.3,

group.missing.thresh=NA,

log2transform=FALSE,medcenter=FALSE,znormtransform=FALSE,quantile_norm=FALSE,lowess_norm=FALSE,madscaling=FALSE,missing.val=0,

networktype="complete", summary.na.replacement="none",samplermindex=NA,

net_node_colors=c("yellow", "green"), net_node_shapes=c("rectangle","circle"),net_edge_colors=c("red","blue"),net_legend=FALSE, netrandseed =555,num_nodes=6)

Output:

1. Rda files for correlations
2. Stage 1 folder: Pre-processing results
3. Stage 2 folder:
   1. correlation_matrix.txt -> pairwise correlations of all m/z features
   2. correlation_pvalues.txt -> p-values for all pairwise correlations
   3. correlation_FDR.txt -> FDR adjusted p-values
   4. significant_correlations_targeted_matrix_mzlabels.txt -> significant correlations with targeted features. The targeted features are represented in rows. Each feature is labeled as the combination of “mz_RetentionTime”.
   5. significant_correlations_targeted_matrix_rowcolnumlabels.txt -> significant correlations with targeted features. The targeted features are represented in rows (X) and all other features are presented in columns (Y). Each feature is labeled by the row or column number.
   6. targetednetwork_plot.pdf - > PDF with network plots (row/column labels and mz_RT labels)
   7. network_Cytoscape_format_maxcor4_mzlabels.gml -> Network file in GML format that can be used as input for Cytoscape. The nodes are labeled by “mz_RetentionTime” combination
   8. network_Cytoscape_format_maxcor4_rowcolnumlabels.gml -> Network file in GML format that can be used as input for Cytoscape. The nodes are labeled by row and column numbers

**3. Opening GML files in Cytoscape:**

1. Open Cytoscape
2. Go to File -> Import -> Network -> File
3. Browse to the location of the output from MetabNet
4. Load the GML file
5. Go to “Layout” on the toolbar and select “Apply Preferred Layout”

Example:


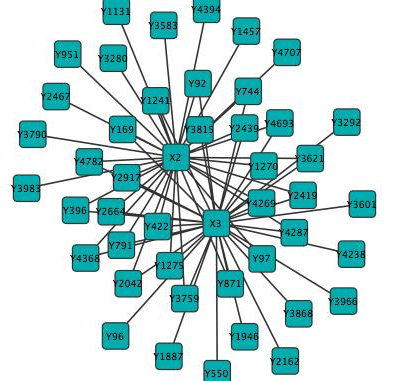


Note: X2 and X3 correspond to the row 2 and 3 in the significant correlations files. And, the Y nodes correspond to the column numbers.
