# Supplementary material for: MetabNet: An R Package for Metabolic Association Analysis of High-Resolution Metabolomics Data
Source: Front Bioeng Biotechnol. 2015 Jun 11;3:87. doi: 10.3389/fbioe.2015.00087 (PMC4464066; doi:10.3389/fbioe.2015.00087)
Supplement: Supplementary file 2 [file table_1.docx]

**Supplementary Results**

**Supplementary Table 1: Metabolites significantly associated (FDR < 0.05) with choline (*m/z* 104.1062) in 50 marmoset samples.**

**Supplementary Table 1: Metabolites significantly associated (FDR < 0.05) with choline (*m/z* 104.1062) in 50 marmoset samples (continued).**

**Supplementary Table 1: Metabolites significantly associated (FDR < 0.05) with choline (*m/z* 104.1062) in 50 marmoset samples (continued).**

**Supplementary Table 1: Metabolites significantly associated (FDR < 0.05) with choline (*m/z* 104.1062) in 50 marmoset samples (continued).**
